# Generating synthetic CEM from low-energy images using deep learning: A future without contrast media? A proof-of-concept study

**DOI:** 10.1186/s41747-026-00681-7

**Published:** 2026-03-16

**Authors:** Konstantinos Zormpas-Petridis, Reza Kalantar, Ludovica Iaccarino, Matteo Mancino, Gianluca Franceschini, Paola Clauser, Valentina Longo, Evis Sala, Paolo Belli, Anna D’Angelo

**Affiliations:** 1 Fondazione Policlinico Universitario Agostino Gemelli IRCCS, Rome, Italy; 2 Università Cattolica del Sacro Cuore, Rome, Italy; 3 Computational Pathology and Spatially-Integrated Omics GSTeP Facility, Fondazione Policlinico Universitario Agostino Gemelli IRCCS, Rome, Italy; 4 The Institute of Cancer Research, London, UK; 5 Royal Marsden Hospital, London, UK; 6 Department of Biomedical Imaging and Image-Guided Therapy, Medical University of Vienna, Vienna, Austria

**Keywords:** Artifacts, Artificial intelligence, Contrast-enhanced mammography, Contrast media, Deep learning

## Abstract

**Objective:**

We used deep learning to generate synthetic, resembling in appearance, iodine-enhanced, mammograms from low-energy contrast-enhanced mammography (CEM) images.

**Materials and methods:**

We retrospectively selected 140 CEM examinations. We trained a two-dimensional cycle-generative adversarial network on 390 images in 100 patients (195 breasts; 102 positive and 93 negative for lesion detection) using paired low-energy and iodine-enhanced images as input and output, respectively. We validated our model in 40 test patients (63 breasts; 37 positive and 26 negative for lesion detection) by calculating the contrast-to-noise ratio (CNR) for low-energy, synthetic, and clinical iodine-enhanced images and the mean absolute error (MAE) and similarity index metric (SSIM) between clinical and synthetic iodine-enhanced images regarding their changes from low-energy. Three radiologists scored (a-to-d) the test set images for background parenchymal enhancement (BPE) and lesion detection (yes/no) on clinical and synthetic images. The presence of artifacts was reported on all images.

**Results:**

We observed a high correlation between clinical and synthetic iodine-enhanced images regarding their changes from low-energy: MAE, *r* = 0.99; SSIM, *r* = 0.80. CNR was -0.015/-0.16 ± 0.23/0.05 (mean ± standard deviation) for clinical/synthetic, respectively. A “halo” artifact present in above 50% of the clinical iodine-enhanced images was corrected in the synthetic ones. On synthetic images, BPE (scores a–b *versus* c–d) was 85.8% accurate. Lesion detection accuracy was 89.4% and 79.4%, sensitivity 87.4 and 72.1%, and specificity 92.3% and 90.0% for clinical and synthetic images, respectively.

**Conclusions:**

Deep learning holds the potential to generate synthetic iodine-enhanced mammograms from low-energy images.

**Relevance statement:**

Radiologists could perform some clinical tasks, such as lesion detection and BPE estimation on synthetic iodine-enhanced images, without contrast injection.

**Key Points:**

- Our deep learning model generated synthetic iodine-enhanced images that visually resembled the clinical iodine-enhanced images.
- Radiologists could use the synthetic images to perform clinical tasks, such as lesion detection and BPE evaluation.
- Our model can improve image quality by removing common artifacts, including the breast-in-breast (halo).
- Our method is a way to combine the benefits of CEM while sparing the need for contrast media.

**Graphical Abstract:**

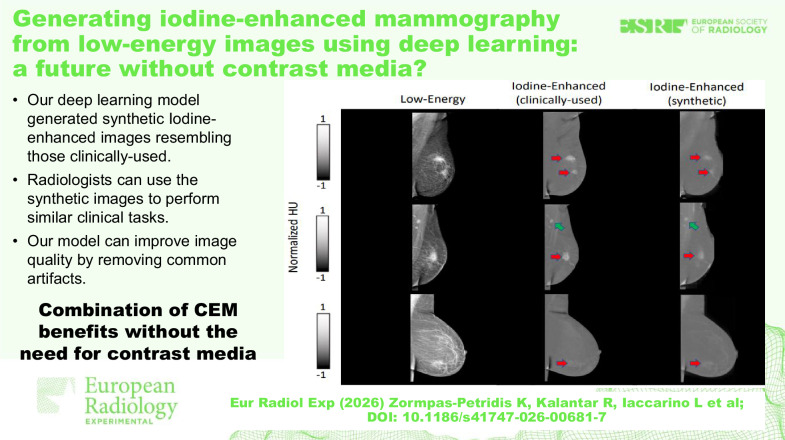

## Background

Contrast-enhanced mammography (CEM) is an increasingly popular modality in breast imaging, particularly for lesion detection and cancer diagnosis [1]. CEM uses iodine-based contrast agents to visualize abnormalities and vascularization, contrary to conventional digital mammography (DM) and digital breast tomosynthesis, which rely solely on attenuation changes between fat, fibroglandular breast tissue, and lesions [2]. CEM can serve as an alternative to contrast-enhanced breast magnetic resonance imaging (MRI), which also uses contrast media—however, gadolinium-based—to visualize lesions with abnormal vascularization, but has longer examination times and higher costs [3, 4]. These features position CEM as a valuable adjunct to existing breast imaging modalities, contributing to comprehensive and efficient breast cancer diagnosis and management.

In this context, CEM demonstrates distinct advantages for screening intermediate-risk women (15%–20% lifetime risk), such as those with a prior history of breast cancer, women diagnosed with high-risk (B3) lesions, as well as those with dense breast tissue or assessing women with inconclusive image findings at DM and/or ultrasound [5]. Extensive data demonstrate that CEM shows significant improvement in sensitivity and specificity compared to DM (with or without ultrasound), in particular in patients with dense breast tissue, to which CEM seems substantially unaffected [1]. Furthermore, the sensitivity of CEM is similar to that of MRI, while potentially being more specific and requiring less examination time by the radiologist, as a standard examination consists of eight images, compared to the several hundred from MRI [4, 6].

Despite its clear benefits, the use of contrast media in CEM introduces certain limitations and considerations. First, contrast media administration is associated with the risk of adverse reactions, including allergic reactions, nephrotoxicity, and anaphylaxis [7–9]. The administration of iodine-based contrast media is not always indicated in women with known allergies to the contrast component, reduced renal function, and hyperthyroidism. Patient selection and prescreening for contraindications to contrast administration are essential to mitigate these risks effectively. Additionally, intravenous contrast injection may be discomforting for some patients, and the interpretation of CEM images may be partially influenced by artifacts related to contrast administration [10]. Despite the low levels of radiation exposure, CEM can increase in radiation dose levels by 20–80% compared to DM, depending on device settings and breast thickness [1].

During CEM acquisition, low-energy and high-energy images per breast and per view are obtained in succession, approximately 2 min after contrast injection and within 10 min [1]. The low-energy image is considered equivalent to standard DM, while the high-energy image is used in postprocessing to generate the recombined or iodine-enhanced image that shows possible areas of contrast enhancement.

To alleviate the need for using contrast agents, in this proof-of-concept study, we hypothesized that deep learning could artificially generate synthetic iodine-enhanced images directly from low-energy images. Architectures inspired by U-Net [11] or generative adversarial networks (GAN) [12] have been successful in capturing image features at multiple spatial resolutions and utilizing them to generate realistic contrast in applications such as image denoising, style transfer, and image reconstruction. We trained a cycle-GAN [13] model to generate synthetic iodine-enhanced images for breasts with or without malignancy. We then tested the feasibility of utilizing synthetic iodine-enhanced images to achieve equivalent performance in routine clinical tasks such as the calculation of the background parenchymal enhancement (BPE) score and lesion detection in a holdout test set.

## Materials and methods

### Population

This retrospective study was approved by the local Institutional Review Board (ID 6921) of Fondazione Policlinico Universitario Agostino Gemelli IRCCS (FPG), Rome, Italy.

We analyzed CEM examinations performed at FPG between April 1, 2021, and August 1, 2023. Inclusion criteria included adult patients (age > 18 years) who underwent CEM as a second-level imaging modality. Patients were eligible if CEM was performed: (i) for further assessment after equivocal findings on mammography and ultrasound; (ii) for local staging of disease following a biopsy-confirmed diagnosis of malignancy, *i.e*., Breast Imaging Reporting and Data System [BI-RADS] 6 [14], or (iii) as part of high-risk screening in individuals with hereditary/familial predisposition to breast cancer. In cases where CEM identified lesions classified as BI-RADS 3 or higher, inclusion required either a minimum of 12 months of clinical and imaging follow-up or histopathological confirmation via biopsy. Exclusion criteria included CEM performed on breasts with a history of surgery and/or radiation therapy, as these interventions may cause tissue alterations that could confound image interpretation and analysis.

The reference standard (ground truth) for lesion characterization was either histopathological confirmation via biopsy (102 patients) or clinical and imaging follow-up of at least 12 months (38 patients). BPE was visually assessed on the clinical recombined/iodine-enhanced images by one of five board-certified breast radiologists, each with at least six years of experience in breast imaging. The final study cohort included 140 female patients aged 60 ± 11 years (mean ± standard deviation).

### CEM protocol

CEM was performed using the Senographe Pristina^TM^ (GE HealthCare). For each patient, low-energy and high-energy CEM images were acquired in succession for both breasts, in craniocaudal (CC) and mediolateral oblique (MLO) views, starting with the affected breast, as per internal protocol. The images were obtained approximately 2 min after the intravenous administration of iodine-based contrast agent (Omnipaque 350, 350 mgI/mL, GE HealthCare). The high-energy images were utilized to compute the recombined/iodine-enhanced images used in the clinical evaluation.

Images were randomly split into a training set (100 patients, 390 paired images) and a testing set (40 patients, 96 paired images). More details can be found on Table 1.

**Table 1:** Distribution of the per-breast lesion detection (‘positive’ for BI-RADS 2 to 5 and ‘negative’ for BI-RADS 1) and per-patient BPE ground-truth scores in the training and test sets of our overall dataset

Clinical task	Training set	Testing set
Lesion detection	195 breasts in 100 patients	63 breasts in 40 patients
Positive	52.3% (102/195)	58.7% (37/63)
Negative	47.7% (93/195)	41.3% (26/63)
BPE assessment	100 patients	40 patients
a (minimal)	50.0% (50/100)	55% (22/40)
b (mild)	37.0% (37/100)	37.5% (15/40)
c (moderate)	9.0% (9/100)	7.5% (3/40)
d (marked)	4.0% (4/100)	0.0% (0/40)

### Deep learning framework

#### Patch-based training strategy and image preprocessing

The original image resolution was either 2,850 × 2,394 pixels or 2,294 × 1.914 pixels, depending on the acquisition settings. A common strategy to accommodate such large images in the input size of deep neural networks is to downsample them [12]. To maximize the diversity of input data and mitigate the loss of critical anatomical detail that can occur with extensive downsampling, here we adopted a patch-based training strategy instead [13]. This approach also served as an effective form of data augmentation. During training, random patches of size 128 × 128 pixels were extracted from the resampled training images.

To ensure that only informative regions were included and to avoid patches consisting solely of background, we generated a breast mask for each image using Otsu thresholding, followed by the extraction of the minimum bounding box encompassing the breast tissue. Given the high spatial resolution of CEM images, we downsampled the images by a factor of four prior to patch extraction. This downsampling allowed each patch to cover a broader field-of-view, increasing the likelihood of capturing complete anatomical structures within individual patches while retaining high resolution [15]. At full resolution, some lesions would exceed the receptive field of the network, making it difficult for the model to learn both the local characteristics of lesions and their global context.

All images were resampled to a uniform pixel spacing of 1.0 × 1.0 mm² using bilinear interpolation to standardize spatial resolution across the dataset. Intensity values were empirically truncated to the range of 1,800–2,900 for low-energy images and 1,700–2,400 for iodine-enhanced images to exclude outlier values and enhance contrast normalization based on the distribution observed in our dataset. Subsequently, the intensity values were linearly scaled to the range of -1.0 to 1.0 to reduce dynamic range and facilitate stable training of the cycle-GAN model.

#### Cycle-GAN

We developed a two-dimensional (2D) cycle-GAN deep neural network inspired by a previously published three-dimensional architecture applied on unpaired data for non-contrast computed tomography synthesis [13]. Cycle-GAN is primarily used for image style transfer and image generation. Images from one domain (*A*) are reconstructed to the distribution of a different domain (*B*), and the process is repeated from domain *B* to *A* for spatial consistency. Therefore, the network architecture requires two generators and two discriminators. In this study, we used a supervised learning approach on 100 patients by using corresponding paired data of low-energy and iodine-enhanced images as input and output (ground-truth), respectively.

We used a Res-Net model for the generator [16], with one convolutional encoding block (2DReflectionPadding-Conv2D-InstanceNorm-Relu), two down-convolution blocks (StridedConv2D-InstanceNorm-Relu), nine residual units, two up-convolutional blocks (StridedTransposedConv2D-InstanceNormRelu), and Tanh activation function for the final layer. The discriminator included three down-convolutional blocks (StridedConv2D-InstanceNorm-LeakyRelu), one 2D convolutional layer, and a sigmoid activation function for the last layer. We focused on generating realistic contrast by utilizing a perceptual-based cycle-loss with equal weighting between the mean-absolute-error and the structural similarity index (SSIM), a least-squares error discriminator loss, and a lambda loss term of ten in the overall identity loss.

The network was trained using an NVIDIA RTX5000 GPU for approximately 6,000 iterations with a batch size of 20. For both the generators and discriminators, the Adam optimizer and a learning rate of 2 × 10^-4^ were utilized. All the code was implemented in Python (version 3.10.8) using the TensorFlow-gpu (version 2.10.0) and Keras libraries for training, and the MONAI framework for data preprocessing [17].

#### Inference

To synthesize the final generated full-size synthetic iodine-enhanced images, a dense sliding window algorithm with an arbitrary stride of 16 pixels (~ 88% overlap, patch size 128 × 128 pixels) was applied on the low-energy images. For overlapped regions, intensity averaging was applied.

### Quantitative evaluation

We performed a quantitative evaluation of the similarity between the network-generated synthetic iodine-enhanced images and the clinical iodine-enhanced images (considered as ground truth) using data from 40 test patients, comprising a total of 96 images. Given that the network was trained to predict relative image contrast rather than absolute intensity values, direct comparison of pixel intensities between synthetic and clinical images was not performed. Instead, similarity was assessed by evaluating the contrast changes from the corresponding low-energy images to both the synthetic and clinical iodine-enhanced images.

We computed the mean absolute error (MAE) and the SSIM between the low-energy and clinical iodine-enhanced images, as well as between the low-energy and synthetic images. The correlation between these two sets of measurements was then evaluated using Pearson’s correlation coefficient to assess the consistency in contrast change patterns.

In addition, we calculated the contrast-to-noise ratio (CNR) for each image type: low-energy, synthetic iodine-enhanced, and clinical iodine-enhanced. An expert radiologist with more than six years of experience in breast imaging (A.D’A; R1) manually annotated regions of fibroglandular tissue (appearing brighter) and adipose tissue (appearing darker) on the low-energy images, carefully excluding enhancing areas potentially associated with underlying lesions. These annotation masks were subsequently transferred to the corresponding clinical and synthetic iodine-enhanced images. CNR values were then computed using the following formula:

$${\mathrm{CNR}}=\frac{\left|{{\mathrm{mean}}}_{{\mathrm{adipose}}}-{{\mathrm{mean}}}_{{\mathrm{fibroglandular}}}\right|}{{{\mathrm{std}}}_{{\mathrm{adipose}}}+{{\mathrm{std}}}_{{\mathrm{fibroglandular}}}}$$

where mean_adipose_ and mean_fibroglandular_ are the average value of the annotated dark areas of adipose tissue and bright areas of fibroglandular tissue, respectively, while std is the standard deviation of the values of the same areas.

### Qualitative evaluation

To evaluate the clinical utility of the synthetic images generated by our model, we assessed the diagnostic performance of three radiologists specialized in breast imaging. The radiologists had 6 years (A.D’A.: R1), 3 years (V.L.: R2), and 2 years (L.I.: R3) of experience, respectively. Their performance was measured in two key clinical routine tasks: the evaluation of BPE and the identification of lesions on both synthetic and clinical iodine-enhanced images.

The reader study was conducted in two separate sessions, each involving either synthetic or clinical images, presented in a randomized and double-blinded order. The readers had no information regarding biopsy results or the reason for CEM examination, and did not have access to any previous imaging studies. For each anonymized patient case, the radiologists assessed BPE according to the BI-RADS classification [14], assigning a BPE score from a to d. These BPE scores were subsequently grouped into minimal–mild (a or b) *versus* moderate–marked (c or d) to obtain more stable estimates [18]. However, for completeness, we also computed the quadratic-weighted κ, as it penalizes large ordinal disagreements more heavily, reflecting the clinical interpretation of BPE categories where adjacent ratings (*e.g*., b *versus* c) are less critical than extreme discordances (a *versus* d). 95% confidence intervals were obtained by 1,000-sample bootstrap resampling at the case level. We report the full confusion matrices for BPE categories (a, b, c, d), comparing synthetic image scores to the corresponding clinical ground-truth scores in the Supplementary material.

In addition, radiologists evaluated each image for the presence of lesions, regardless of the degree of radiological suspicion, categorizing them as positive (in the case of BI-RADS 2–5 findings) or negative (for BI-RADS 1 findings). The readers were instructed to report any findings assessed to be false enhancement patterns or artificial structures (“hallucinations”). To minimize recall bias, the two sessions were separated by a minimum interval of two weeks, ensuring that no radiologist assessed synthetic and clinical images from the same patient in the same session. The study included a total of 40 test patients, encompassing 63 breasts, of which 37 were positive and 26 were negative for detected lesions.

During the evaluations, the “halo artifact”—also referred to as the “breast-in-breast” artifact—was identified by the readers in the majority of clinical iodine-enhanced images. This artifact, well documented in the literature [19], arises from non-uniform breast thickness and scattered radiation, leading to spurious enhancement at the breast periphery.

The accuracy, sensitivity, and specificity scores for the detection of lesions were calculated for each reader using the following formulae:

$${\hskip -30pt}{\mathrm{Accuracy}}=\frac{{\mathrm{TP}}+{\mathrm{TN}}}{{\mathrm{TP}}+{\mathrm{TN}}+{\mathrm{FP}}+{\mathrm{FN}}}, {\mathrm{Sensitivity}}=\frac{{\mathrm{TP}}}{{\mathrm{TP}}+{\mathrm{FN}}}, {\mathrm{Specificity}}=\frac{{\mathrm{TN}}}{{\mathrm{TN}}+{\mathrm{FP}}}$$

where TP is true positive, TN is true negative, FP is false positive, and FN is false negative.

### Statistical analysis

Significant correlations were determined using the Pearson correlation coefficient. The “Wilcoxon” function in the SciPy Python package (version 1.9.3) was used to assess any significant differences, assuming a two-sided alternative hypothesis. Reader assessments were treated as independent evaluations. BPE and lesion detection were evaluated as separate endpoints with calculated *p*-values corrected for multiple comparisons by using the Benjamini-Hochberg procedure, and a *p*-value lower than 0.05 was chosen to indicate significance.

## Results

The patient data were randomly split into a training set (100 patients, 390 paired images) and a testing set (40 patients, 96 paired images). Age of the two groups were 59 ± 11 years and 62 ± 10 years (mean ± standard deviation), respectively. More details can be found in Table 1.

### Synthetic image generation

**Fig. 1:**
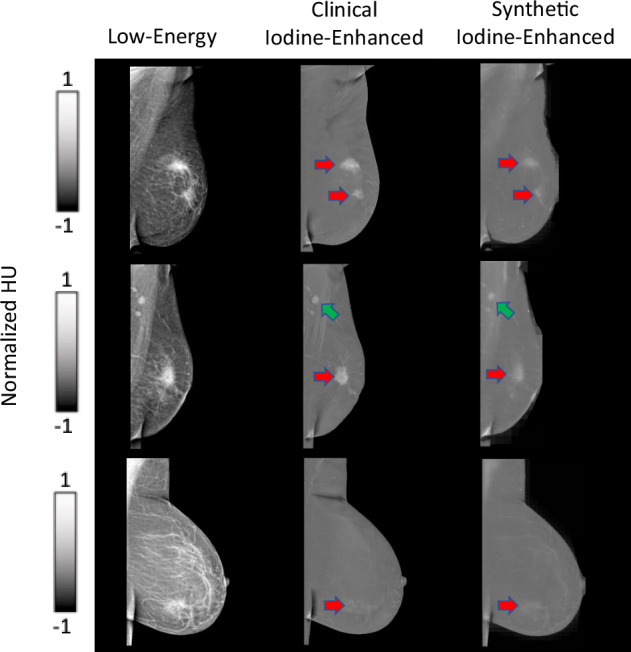
Representative examples of three cases with detected lesions, specifically BI-RADS 5 (red arrows). Note that the axillary lymph nodes are retained in the synthetic iodine-enhanced images (green arrows)

The neural network achieved convergence after approximately 4,500 training iterations. Visual assessment by the expert radiologists confirmed that the synthetic iodine-enhanced images closely resembled the clinical iodine-enhanced images in terms of both overall appearance and contrast. Notably, the neural network effectively suppressed the signal intensity of adipose tissue while preserving enhancement in regions of clinical interest, such as suspected lesions, as illustrated in Fig. 1 (red arrows). In addition, key anatomical features, including lymph nodes (Fig. 1, green arrows) and vascular structures, were accurately retained in the synthetic images.

**Fig. 2:**
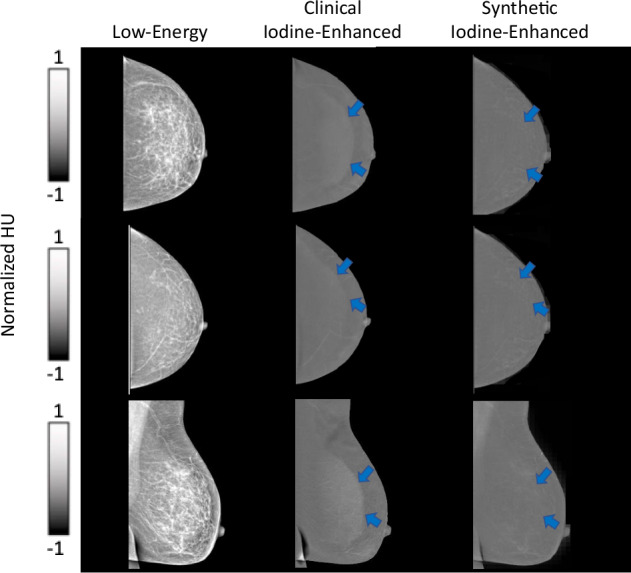
Representative examples of cases presenting with no lesions. Note the “halo” artifact present in the clinical iodine-enhanced images that has been removed in the synthetic iodine-enhanced images (blue arrows)

The C-shaped “halo” artifact in the breast periphery was observed in over 50% of the clinical iodine-enhanced images and was corrected by our model in all the synthetic images. This correction is demonstrated in Fig. 2 and is attributed to the bidirectional style transfer inherent to the cycle-GAN architecture, which enables learning from both image domains (*A* → *B* and *B* → *A*). The reconstruction capability of the model allows for effective contrast transfer between modalities while preserving the underlying spatial anatomy. Importantly, no false enhancement patterns or artificial structures (“hallucinations”) were detected in any of the synthetic images. Representative examples are presented in Figs. 1 and 2.

### Quantitative evaluation

The synthetic iodine-enhanced, images demonstrated a strong similarity to the clinical iodine-enhanced images in terms of their intensity changes relative to low-energy images. Specifically, the synthetic images showed a high correlation with the clinical iodine-enhanced images, with MAE reaching *r* = 0.99, and SSIM of 0.80, indicating strong structural alignment.

CNR analysis was conducted for annotated regions of adipose tissue and fibroglandular tissue. This analysis revealed that the synthetic images effectively reduced the enhancement of fibroglandular tissue, aligning closely with the CNR levels observed in the clinical iodine-enhanced images. After normalization, the mean CNR values were 0.66 for low-energy images, -0.015 for clinical iodine-enhanced images, and -0.169 for synthetic images.

The intensity distribution within the annotated regions showed reduced variability in the synthetic images compared to the clinical iodine-enhanced images. Specifically, the standard deviation of CNR values was 0.59 for low-energy, 0.24 for clinical iodine-enhanced, and 0.05 for synthetic images.

A slight trend was observed in which synthetic images underestimated intensity values compared to clinical iodine-enhanced images. However, in adipose tissue regions, synthetic images showed slightly higher mean values than clinical iodine-enhanced images after normalization (low-energy = 0.607, clinical iodine-enhanced = -0.096, synthetic = -0.087), although the difference was minor.

**Fig. 3:**
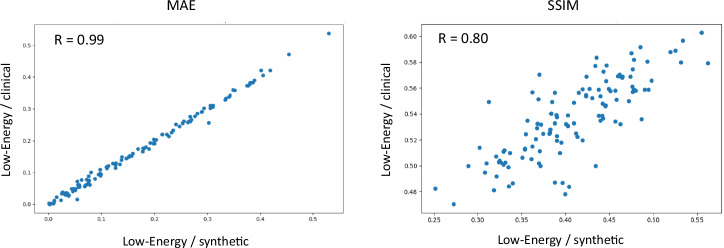
Quantitative evaluation of the similarity of the changes from low-energy to clinical iodine-enhanced and synthetic iodine-enhanced images. MAE, Mean absolute error; SSIM, Structural-similarity-index metric

**Fig. 4:**
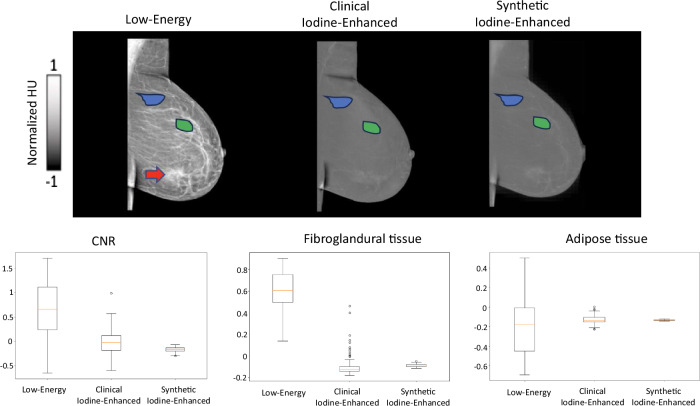
Quantitative evaluation of CNR for annotated areas of fibroglandular tissue (green region) and adipose tissue (blue region) and their respective normalized values for synthetic and clinical iodine-enhanced images. Signal-enhancing areas on low-energy images, potentially associated with underlying lesions, were excluded (red arrow)

These results are detailed in Table 2 and visualized in Figs. 3 and 4.

**Table 2:** Summary of results: quantitative evaluation

Metrics	Images		
	Low-energy	Clinical iodine-enhanced	Synthetic
CNR	0.66 ± 0.59	-0.015 ± 0.24	-0.169 ± 0.05
Adipose tissue	0.607 ± 0.17	-0.096 ± 0.09	-0.087 ± 0.01
Fibroglandular tissue	-0.181 ± 0.28	-0.127 ± 0.04	-0.132 ± 0.004

Data are given as mean ± standard deviation

### Qualitative evaluation

The accuracy of BPE assessments, compared to the reference clinical scores, was 90%, 77.5%, and 90% for the three readers, R1, R2, and R3, respectively (data generating these ratios are presented in Supplementary Table S1). A minor trend toward overestimation of BPE in the synthetic images was observed due to slightly higher values in areas with a lot of adipose tissue in the low-energy images, resulting in a mean quadratic-weighted κ of 0.43 [0.38–0.52, 95% confidence interval: 0.199–0.612] for all BPE categories. Full confusion matrices comparing synthetic image scores to the corresponding clinical ground-truth scores are presented in Supplementary Tables S2–S4.

Following this evaluation, the radiologists also identified abnormal findings (BI-RADS categories 2–5) and negative images (BI-RADS category 1) in both the synthetic and clinical iodine-enhanced images. Using the clinical iodine-enhanced images, the three readers achieved an overall 89.4% mean accuracy, 87.4% mean sensitivity, and 92.3% mean specificity, while for the synthetic images, the corresponding results were 79.4%, 72.1%, and 90.0%. The readers, in agreement with the quantitative evaluation, observed a general minor trend of lower average image pixel intensity of the suspected lesions in the synthetic images compared to the clinical iodine-enhanced images. The readers reported no false enhancement patterns or artificial structures (“hallucinations”). Notably, there was no case in which all three radiologists failed to detect a lesion on the synthetic images; in every instance, at least one reader identified the lesion correctly.

**Fig. 5:**
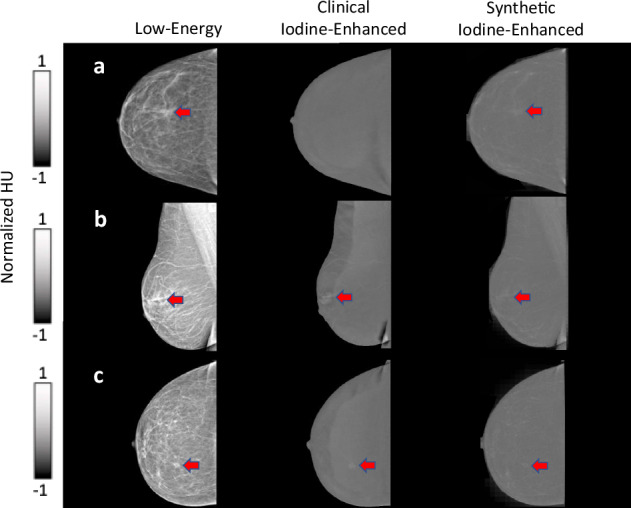
Representative cases illustrating differences in reader performance between clinical iodine-enhanced and synthetic images. In all panels, a suspicious lesion (red arrow) is present. **a** A case in which the lesion was missed by all three readers on the clinical iodine-enhanced but correctly identified by all three on the synthetic image. **b**, **c** Cases in which the lesion was identified by all three readers on the clinical iodine-enhanced images but detected by only one of three readers on the synthetic images. The lesion was present in the synthetic images, but was more difficult to distinguish from the background

Detailed results are presented in Table 3. Cases where there were notable differences in the reader examination of the clinical iodine-enhanced and synthetic images are presented in Fig. 5.

**Table 3:** Summary of results: qualitative reader evaluation study

BPE Minimal/mild (a or b), moderate/marked (c or d)			
Number of patients	R1	R2	R3
40	36/40 = 90%	36/40 = 90%	28/40 = 77.5%
**Lesion detection** **Yes/No**			
**Number of breasts**	**Metric**	**Clinical iodine-enhanced images**	**Synthetic images**
Positive = 37 Negative = 26	Accuracy		
	R1	57/63 = 90.5%	49/63 = 77.8%
	R2	56/63 = 88.9%	51/63 = 81.0%
	R3	56/63 = 88.9%	50/63 = 79.4%
	Sensitivity		
	R1	34/37 = 91.9%	26/37 = 70.3%
	R2	32/37 = 86.5%	28/37 = 75.7%
	R3	31/37 = 83.8%	26/37 = 70.3%
	Specificity		
	R1	23/26 = 88.5%	23/26 = 88.5%
	R2	24/26 = 92.3%	23/26 = 88.5%
	R3	25/26 = 96.2%	24/26 = 92.3%

## Discussion

Our proof-of-concept study presented a model that successfully generated synthetic, resembling in appearance iodine-enhanced, CEM images directly from low-energy inputs, demonstrating that synthetic contrast images could support routine clinical tasks without the need for injecting contrast agents. The synthetic images achieved high structural similarity to the clinical iodine-enhanced images, with a mean SSIM of 0.80 and comparable mean CNR values, indicating effective suppression of fibroglandular tissue signal similar to that seen in the clinical images. The smaller standard deviation of the CNR values suggests that the synthetic images contained fewer outliers and exhibited more consistent intensities within the tissue. We demonstrated good performance on calculating the BPE score and lesion detection with 85.8% and 79.4% average accuracy, respectively. While diagnostic performance was higher with the clinical iodine-enhanced images, the synthetic images provided comparable specificity, moderate sensitivity, and accuracy, particularly in the identification of negative cases. Moreover, our cycle-GAN model showed potential in further improving clinical image quality by completely removing common artifacts, such as the “halo artifact” which can obscure a proportion of the image.

There is a significant population of intermediate-risk breast cancer patients, including women with dense breasts, family history of breast cancer, high-risk lesions, or prior breast cancer, who would benefit from personalized screening programs [5]. However, widespread adoption of CEM in screening is currently limited by challenges associated with iodinated contrast agents, such as production and storage issues, side effects, and patient discomfort. Moreover, although the levels of radiation exposure of CEM are low (~ 1.2 mSv per exam), it could still be a further consideration in cases of repeated screening exams, acquisition of only low-energy images (~ 0.4 mSv per exam) would be preferable. Our cycle-GAN model retained the high specificity of CEM, which is one of the main considerations in a screening setting and a key advantage over MRI, while showing good sensitivity. Additionally, despite the increasing popularity of MRI for high-risk patients and women with extremely dense breasts [20], as is evident by the DENSE trial and the EA1411 ECOG-ACRIN study [21], CEM offers an alternative that addresses the high costs, patient comfort, and limited availability associated with MRI screening.

Understanding the underlying mechanisms that allow the neural network to generate iodine-enhanced images is crucial, particularly since the network cannot directly predict the effects of contrast media on breast tissue. Although difficult for human observers, the network likely captures unique characteristics, such as the texture and shape of suspicious findings, and is able to distinguish them from the enhanced signal of adipose tissue even in low-energy images. This approach of image generation integrates seamlessly into the existing radiological assessment workflow, ensuring transparency by mitigating the common “black-box” properties associated with prediction algorithms, thereby enabling easier clinical translation. It can also be used to complement lesion detection and BPE calculation algorithms, which are applied directly to low-energy images or DM images.

Beyond overall performance metrics, we also carefully reviewed the cases in which radiologists failed to identify lesions on the synthetic images. We observed that the missed detections did not concern a specific abnormality type but were consistently attributable to two trends noted earlier: (i) slightly higher average pixel intensity in adipose tissue; and (ii) slightly lower lesion signal relative to clinical iodine-enhanced images. Together, these effects reduced lesion conspicuity in certain cases, although the lesions were in fact present on the synthetic images when examined side-by-side with the clinical iodine-enhanced counterparts. Importantly, during the qualitative study, there was no case in which all three radiologists failed to detect a lesion on the synthetic images; in every instance, at least one reader identified the lesion correctly. Conversely, one lesion was missed by all three readers on the clinical iodine-enhanced images but detected by all three on the synthetic image.

This study was conducted as proof-of-concept and has limitations. Our dataset used was monocentric, using a single vendor scanner, and the size of the test set (40 patients; 73 breasts) is not sufficient to establish clinical equivalence considering differences among different equipment [22, 23]. Also, achieving at least equivalent sensitivity between synthetic and clinical iodine-enhanced images is necessary for clinical integration. To address these points, we plan to fine-tune our model on larger, multicenter datasets to improve robustness and capture greater variability. Additionally, we will explore the use of stable-diffusion models [24], which have shown considerable promise in generating realistic and accurate contrast, being at times superior and easier to train than cycle-GAN architectures [25]. Further refinement using perceptual loss functions, leveraging deep feature image representation, may also enhance image quality over traditional methods like SSIM. Considering that MAE and SSIM do not capture perceptual texture details or subtle diagnostic features such as microcalcifications, we will incorporate advanced perceptual similarity metrics (*e.g*., Learned Perceptual Image Patch Similarity [26] or Fréchet Inception Distance [27]) to more comprehensively evaluate the fidelity of synthetic images. In future work, we will extend the qualitative evaluation by distinguishing between benign and malignant lesions, rather than relying solely on identifying lesion presence. This will enable a more clinically meaningful assessment of diagnostic performance, particularly in evaluating the potential of synthetic images to support lesion characterization, risk stratification, and clinical decision-making. We also aim to apply our method directly to DM images, as low-energy images in CEM are inherently acquired after contrast administration. Although low-energy images are generally regarded as equivalent to DM [1], extending our approach to true conventional DM will enable us to assess the feasibility and performance of synthetic iodine-enhanced image generation in screening populations, where contrast is not routinely used.

We conclude that deep learning models, such as ours, could generate synthetic, iodine-enhanced images resembling in appearance iodine-enhanced images from low-energy inputs, potentially paving the way for the combination of the benefits of CEM without the need for contrast media, while also improving image quality and reducing radiation exposure. Further improvements and validation of our approach in DM images could have a major impact on personalized breast cancer screening programs for intermediate-risk patients and patients with extremely dense breasts.

## Supplementary information

**Additional file 1: Table S1**. Confusion matrix of background parenchymal enhancement (BPE) scores for Reader R1 compared with the clinical ground-truth. Quadratic-weighted kappa: 0.52 (95% CI 0.224–0.723). **Table S2**. Confusion matrix of background parenchymal enhancement (BPE) scores for Reader R2 compared with the clinical ground-truth. Quadratic-weighted kappa: 0.39 (95% CI 0.13–0.70). **Table S3**. Confusion matrix of background parenchymal enhancement (BPE) scores for Reader R3 compared with the clinical ground-truth. Quadratic-weighted kappa: 0.38 (95% CI 0.14–0.56)

Supplementary tableS2

Supplementary tableS3

Supplementary tableS4 Confusion matrix of background parenchymal enhancement (BPE) scores for Reader R3 compared with the clinical ground-truth. Quadratic-weighted kappa: 0.38 (95% CI 0.14–0.56)

## Data Availability

The datasets used and/or analyzed during the current study are available from the corresponding author on reasonable request.

## Abbreviations

2D: Two-dimensional
BI-RADS: Breast imaging reporting and data system
BPE: Background parenchymal enhancement
CEM: Contrast-enhanced mammography
CNR: Contrast-to-noise ratio
DM: Digital mammography
GAN: Generative adversarial network
SSIM: Structural similarity index metric
