# Supplementary material for: Generating synthetic CEM from low-energy images using deep learning: A future without contrast media? A proof-of-concept study
Source: Eur Radiol Exp. 2026 Mar 16;10:31. doi: 10.1186/s41747-026-00681-7 (PMC12992836; doi:10.1186/s41747-026-00681-7)
Supplement: Supplementary file 1 — Additional file 1: Table S1. Confusion matrix of background parenchymal enhancement (BPE) scores for Reader R1 compared with the clinical ground-truth. Quadratic-weighted kappa: 0.52 (95% CI 0.224–0.723). Table S2. Confusion matrix of background parenchymal enhancement (BPE) scores for Reader R2 compared with the clinical ground-truth. Quadratic-weighted kappa: 0.39 (95% CI 0.13–0.70). Table S3. Confusion matrix of background parenchymal enhancement (BPE) scores for Reader R3 compared with the clinical ground-truth. Quadratic-weighted kappa: 0.38 (95% CI 0.14–0.56) [file 41747_2026_681_MOESM1_ESM.pdf]

Generating synthetic CEM from low-energy images using deep learning: a future without contrast media? A proof-of-concept study

ELECTRONIC SUPPLEMENTARY MATERIAL

**Background parenchymal enhancement**<br>Minimal/mild (a or b), moderate/marked (c or d)				
	**R1**	**R3**	**R2**	**Ground-truth**
Test patient 1	Minimal/mild	Minimal/mild	Moderate/marked	Minimal/mild
Test patient 2	Minimal/mild	Minimal/mild	Moderate/marked	Minimal/mild
Test patient 3	Minimal/mild	Minimal/mild	Minimal/mild	Minimal/mild
Test patient 4	Minimal/mild	Minimal/mild	Moderate/marked	Minimal/mild
Test patient 5	Minimal/mild	Minimal/mild	Moderate/marked	Minimal/mild
Test patient 6	Minimal/mild	Minimal/mild	Moderate/marked	Minimal/mild
Test patient 7	Moderate/marked	Moderate/marked	Moderate/marked	Moderate/marked
Test patient 8	Minimal/mild	Minimal/mild	Minimal/mild	Minimal/mild
Test patient 9	Minimal/mild	Minimal/mild	Minimal/mild	Minimal/mild
Test patient 10	Minimal/mild	Moderate/marked	Moderate/marked	Moderate/marked
Test patient 11	Minimal/mild	Minimal/mild	Minimal/mild	Minimal/mild
Test patient 12	Minimal/mild	Minimal/mild	Minimal/mild	Minimal/mild
Test patient 13	Minimal/mild	Minimal/mild	Minimal/mild	Minimal/mild
Test patient 14	Moderate/marked	Minimal/mild	Moderate/marked	Minimal/mild
Test patient 15	Moderate/marked	Moderate/marked	Moderate/marked	Moderate/marked
Test patient 16	Minimal/mild	Minimal/mild	Minimal/mild	Minimal/mild
Test patient 17	Minimal/mild	Minimal/mild	Minimal/mild	Minimal/mild
Test patient 18	Minimal/mild	Minimal/mild	Minimal/mild	Minimal/mild
Test patient 19	Minimal/mild	Minimal/mild	Minimal/mild	Minimal/mild
Test patient 20	Minimal/mild	Minimal/mild	Minimal/mild	Minimal/mild
Test patient 21	Minimal/mild	Minimal/mild	Minimal/mild	Minimal/mild

Test patient 22	Moderate/marked	Moderate/marked	Moderate/marked	Minimal/mild
Test patient 23	Minimal/mild	Minimal/mild	Minimal/mild	Minimal/mild
Test patient 24	Minimal/mild	Minimal/mild	Minimal/mild	Minimal/mild
Test patient 25	Minimal/mild	Minimal/mild	Minimal/mild	Minimal/mild
Test patient 26	Minimal/mild	Minimal/mild	Minimal/mild	Minimal/mild
Test patient 27	Minimal/mild	Minimal/mild	Minimal/mild	Minimal/mild
Test patient 28	Minimal/mild	Minimal/mild	Minimal/mild	Minimal/mild
Test patient 29	Minimal/mild	Minimal/mild	Minimal/mild	Minimal/mild
Test patient 30	Minimal/mild	Minimal/mild	Moderate/marked	Minimal/mild
Test patient 31	Minimal/mild	Minimal/mild	Minimal/mild	Minimal/mild
Test patient 32	Minimal/mild	Minimal/mild	Minimal/mild	Minimal/mild
Test patient 33	Minimal/mild	Minimal/mild	Moderate/marked	Minimal/mild
Test patient 34	Minimal/mild	Minimal/mild	Minimal/mild	Minimal/mild
Test patient 35	Minimal/mild	Moderate/marked	Minimal/mild	Minimal/mild
Test patient 36	Minimal/mild	Moderate/marked	Moderate/marked	Minimal/mild
Test patient 37	Moderate/marked	Moderate/marked	Moderate/marked	Minimal/mild
Test patient 38	Minimal/mild	Minimal/mild	Minimal/mild	Minimal/mild
Test patient 39	Moderate/marked	Moderate/marked	Moderate/marked	Moderate/marked
Test patient 40	Minimal/mild	Minimal/mild	Moderate/marked	Minimal/mild
**Accuracy**	**36/40**	**36/40**	**28/40**	

Table S1. Confusion matrix of background parenchymal enhancement (BPE) scores for Reader R1 compared with the clinical ground-truth. Quadratic-weighted kappa: 0.52 (95% CI 0.224–0.723)

Background Parenchymal Enhancement (BPE) – Reader R1				
	Synthetic Score a	Synthetic Score b	Synthetic Score c	Synthetic Score d
Ground truth Score a	11	11	1	0
Ground truth Score b	2	9	2	0
Ground truth Score c	0	1	2	1
Ground truth Score d	0	0	0	0

Table S2. Confusion matrix of background parenchymal enhancement (BPE) scores for Reader R2 compared with the clinical ground-truth. Quadratic-weighted kappa: 0.39 (95% CI 0.13–0.70)

Background Parenchymal Enhancement (BPE) – Reader R2				
	Synthetic Score a	Synthetic Score b	Synthetic Score c	Synthetic Score d
Ground truth Score a	13	7	2	1
Ground truth Score b	4	8	1	0
Ground truth Score c	0	0	4	0
Ground truth Score d	0	0	0	0

Table S3. Confusion matrix of background parenchymal enhancement (BPE) scores for Reader R3 compared with the clinical ground-truth. Quadratic-weighted kappa: 0.38 (95% CI 0.14–0.56)

Background Parenchymal Enhancement (BPE) – Reader R3				
	Synthetic Score a	Synthetic Score b	Synthetic Score c	Synthetic Score d
Ground truth Score a	9	12	2	0
Ground truth Score b	2	6	4	1
Ground truth Score c	0	2	1	1
Ground truth Score d	0	0	0	0
