# Supplementary material for: Generating synthetic CEM from low-energy images using deep learning: A future without contrast media? A proof-of-concept study
Source: Eur Radiol Exp. 2026 Mar 16;10:31. doi: 10.1186/s41747-026-00681-7 (PMC12992836; doi:10.1186/s41747-026-00681-7)
Supplement: Supplementary file 4 — Supplementary tableS4 Confusion matrix of background parenchymal enhancement (BPE) scores for Reader R3 compared with the clinical ground-truth. Quadratic-weighted kappa: 0.38 (95% CI 0.14–0.56) [file 41747_2026_681_MOESM4_ESM.docx]

**Table S4.** Confusion matrix of background parenchymal enhancement (BPE) scores for Reader R3 compared with the clinical ground-truth. Quadratic-weighted kappa: 0.38 (95% CI 0.14–0.56)

| **Background Parenchymal Enhancement (BPE) – Reader R3** | | | | |
| --- | --- | --- | --- | --- |
|  | **Synthetic**  **Score a** | **Synthetic**  **Score b** | **Synthetic**  **Score c** | **Synthetic**  **Score d** |
| **Ground truth**  **Score a** | 9 | 12 | 2 | 0 |
| **Ground truth**  **Score b** | 2 | 6 | 4 | 1 |
| **Ground truth**  **Score c** | 0 | 2 | 1 | 1 |
| **Ground truth**  **Score d** | 0 | 0 | 0 | 0 |
